# Autonomy and identity: the role of two developmental tasks on adolescent's wellbeing

**DOI:** 10.3389/fpsyg.2024.1309690

**Published:** 2024-04-10

**Authors:** Wawie D. G. Ruiz, Homer J. Yabut

**Affiliations:** ^1^Department of Psychology, Central Luzon State University, Munoz, Nueva Ecija, Philippines; ^2^Department of Psychology, De La Salle University, Manila, Philippines

**Keywords:** emotional autonomy, identity commitment, psychological wellbeing, adolescent, moderation analysis

## Abstract

Developmental tasks theory suggests that the wellbeing of adolescents can be better understood by considering their developmental tasks, rather than just viewing it as a specific age. This concept has significantly influenced contemporary studies on the transition to adulthood. Study explains that psychosocial developmental tasks involve shifts in an individual's psychological state and social relationships, potentially disrupting their previous equilibrium. Authors stress the importance of forming personal identity and fostering a healthy sense of independence, especially concerning identity and autonomy tasks, as crucial elements for adolescent wellbeing analysis. In line with this, the study focuses on the concurrent and predictive relationship between identity process of commitment, emotional autonomy, and psychological wellbeing among adolescents. Four hundred fifty-four (454) participants with an age ranged from 15 to 19 years (*m* = 17.96) completed surveys measuring emotional autonomy, identity commitment, and psychological wellbeing. The findings revealed that both emotional autonomy and identity commitment predicted psychological wellbeing, but in different ways. The study revealed that emotional autonomy is inversely associated with psychological wellbeing in middle and late adolescents, indicating that their ability to establish independence from their parents or relinquish unrealistic parental expectations may be linked to a decline in their mental health. The results also indicated that identity commitment is moderately and positively related to psychological wellbeing, suggesting that when adolescents have a clear sense of their identity, they may experience greater wellbeing. This finding underscores the importance of encouraging adolescents to explore their values, interests, and goals, as well as providing them with support and guidance throughout the process. Additionally, the findings of the study revealed that emotional autonomy has a negative influence on psychological wellbeing when identity commitment is low or average. Specifically, when individuals have a low level of identity commitment, emotional autonomy significantly predicts lower psychological wellbeing. Similarly, when identity commitment is at an average level, emotional autonomy also has a detrimental effect on psychological wellbeing. Lastly, the study revealed that when individuals have a high level of identity commitment, emotional autonomy does not have a significant impact on psychological wellbeing. In simpler terms, when individuals possess a strong sense of commitment to their identity, their level of emotional autonomy does not play a significant role in influencing their psychological wellbeing.

## Introduction

Adolescence is a stage of development in which significant changes take place, including psychological, physical, and social changes as the adolescent transitions from childhood to adulthood. Throughout this developmental period, it is assumed that these changes can affect the adolescent's wellbeing, which may cause a variety of problem behaviors and negative adjustment outcomes. For instance, in a report by the Department of Health (2020), the remarkable increase in mental health-related deaths among those aged 15–29 provides evidence for the vulnerability of adolescents to experience debilitating psychological problems. In addition, adolescent neurodevelopment is a period of significant brain changes, including alterations in reward/aversive systems, inhibitory control, and emotional processing (Spear, 2013). These changes, combined with the immaturity of the brain, make adolescents more vulnerable to the effects of alcohol and drug abuse, which can lead to long-term behavioral dysfunction (Guerri and Pascual, 2019). Several studies on life satisfaction and wellbeing during adolescence have confirmed this claim and pointed out that at ages 13–20 years, several aspects of adolescents' wellbeing declined (Goswani, 2013; Casas et al., 2014; Gonzales-Carrasco et al., 2016).

While the adolescent period is generally considered a typical part of development involving various changes (World Health Organization, 2019), and is often characterized as a period of turmoil and stress (Casey et al., 2010), it's crucial to comprehend the factors that contribute to this developmental phenomenon. In Western cultures, the decline in wellbeing during this period is seen as a normal part of adolescent development (Stuart and Jose, 2014). However, Park (2004) cross-cultural study demonstrated that the decline in wellbeing, as well as the extent to which contextual and individual factors influence it, can vary significantly in terms of the range and trajectory of change. Despite this, there has been limited research focused on identifying the predictors of adolescent psychological wellbeing (Witten et al., 2019).

The main idea presented in the influential works of Havighurst (1952) suggests that the wellbeing of adolescents can be better understood by considering their developmental tasks, rather than just viewing it as a specific age range from 13 to 19 years old. This concept has greatly impacted contemporary studies on the transition into adulthood (Roisman et al., 2004). Steinberg (2005) explains that psychosocial developmental tasks involve changes in an individual's previous psychological state and social relationships, which can unsettle their previous equilibrium. As emphasized by several authors (Sandu et al., 2011; Avedissian and Alayan, 2021), the formation of personal identity and the establishment of a healthy sense of independence, particularly in relation to tasks surrounding identity and autonomy, are significant factors for adolescents and can be used to analyze aspects of their wellbeing. Despite the common belief that emotional autonomy and identity commitment lead to greater wellbeing, previous research on the concurrent and predictive relationships between these constructs has produced conflicting results and has not been empirically studied in Filipino adolescent samples (Perez, 2012). Therefore, the present study aims to address this gap in the literature by examining whether the interaction of emotional autonomy and identity commitment mitigates the expected decrease in the wellbeing of Filipino adolescents. This study focuses on the concurrent and predictive connections between identity processes, emotional autonomy, and psychological wellbeing among adolescents. In this connection, this present study tries to understand whether emotional autonomy predicts psychological wellbeing (1), assess if identity commitment influences psychological wellbeing (2), and how identity commitment moderates the relationship of emotional autonomy and psychological wellbeing (3).

### The adolescent's wellbeing

Human development has long been concerned with achieving optimal wellbeing. While traditional psychology research has primarily focused on psychopathology, recent years have witnessed the rise of positive psychology, which has introduced new areas of study, including wellbeing and happiness (Henderson and Knight, 2012). Positive psychology views wellbeing as a result of a fulfilling life and has been extensively investigated through empirical research and theoretical advancements. According to Seligman (2003), wellbeing entails seeking positive emotions linked to one's past, present, and future, along with developing personal virtues to attain a purposeful life. Positive emotions, which are closely linked to cognition, play a crucial role in this pursuit, as they can foster broadened, flexible mindsets and contribute to lasting wellbeing (Kiken and Fredrickson, 2017).

Psychological wellbeing is primarily concerned with understanding the structure and dynamics of individual wellbeing, including its causes and consequences, and has been empirically studied from various perspectives. Due to its relevance to other dimensions such as interpersonal ability, levels of life satisfaction, flourishing, and happiness, psychological wellbeing has garnered interest from many fields (Gao and McLellan, 2018; Ryff, 2019). This construct encompasses psychological, social, and subjective dimensions, as well as health-related practices and behaviors that contribute to a positive evaluation of life and the maximization of potential (Ryff, 2014; Ferrari et al., 2015; Lun and Bond, 2016; Friedman et al., 2017; Brim et al., 2019). Several studies have shown that psychological wellbeing is evidence that individuals have developed optimal psychological functioning, which enriches their subjective life experiences. It is also considered a group of factors that encourage people to pursue optimal development (Crous, 2017; Maurya and Ojha, 2017; Bojanowska and Piotrowski, 2019) and is a vital factor in achieving one's optimum potential and meeting expectations in life. Therefore, meeting expectations is often considered a predictor of optimal development and is linked with an increase in overall wellbeing (McDowall, 2016; Reis et al., 2018).

The most recent operationalization of wellbeing is Ryff's (1989) model of psychological wellbeing, which proposes six essential factors: purpose in life, self-acceptance, positive relations with others, personal growth, autonomy, and environmental mastery. Psychological wellbeing is not just the absence of illness or the presence of positive subjective thoughts. However, it includes the mechanisms of healthy functioning and adjustment essential to living optimally (Diaz et al., 2006). In this connection, psychological wellbeing during adolescence plays an integral part in the adaptive development that transcends until adulthood (Gilman and Huebner, 2003; Proctor et al., 2009; Tomyn and Cummins, 2011). An increase in wellbeing serves to protect adolescents against the debilitating psychological problem for the reason that it can stimulate adolescents' ability to think creatively, increase intrapersonal and interpersonal ability, and cultivate inquisitiveness (Proctor et al., 2009). However, several studies stressed that in the early period of adolescent development until the middle years, there is a remarkable decline in wellbeing and that 16 years of age experienced a significant reduction (Tomyn and Cummins, 2010; Casas et al., 2014; Gonzales-Carrasco et al., 2016). It is also pointed out that during this stage, given its multifaceted changes, the ability of adolescents to gain an increased level of wellbeing is jeopardized (Keyes, 2006; Casas et al., 2009; Orkibi et al., 2014).

Research on Filipino psychological wellbeing has explored various factors and dimensions. Villarosa and Ganotice (2018) found that autonomy, environmental mastery, personal growth, positive relations with others, purpose in life, and self-acceptance are key components of psychological wellbeing. In a study among Filipino adolescents, different outcomes are observed in the number of dimensions or factors of psychological wellbeing (Perez, 2012). Interestingly, Filipino adolescents appeared to have difficulty managing their day-to-day affairs as they performed poorly in environmental mastery. Adolescents feel incapable of modifying their situation, may not be aware of the opportunities offered, and may feel powerless when controlling their outside world (Perez, 2012). In the same study, adolescents have low scores for positive relations toward others, which indicates limited close friends, trouble being warm, trusting relationships with others, and concern and openness to others. Adolescents tend not to compromise to maintain significant ties or bonds with others and isolate and frustrate interpersonal relationships (Perez, 2012). It is also highlighted that Filipino adolescents have low average performance in self-acceptance. Adolescents appear discontented about themselves, dissatisfied with past experiences, tend to feel worried about personal characteristics, and generally are different from what they are (Perez, 2012).

That said, adolescence is a crucial developmental period during which various dimensions of psychological wellbeing are established, experienced, and socially and individually differentiated. Several findings from an international perspective support the importance of assessing psychological wellbeing during this period of development (Balcázar-Nava et al., 2008; Medina and Velásquez, 2017). Understanding the psychological wellbeing of young people and the factors that contribute to it can help clarify and define how adolescents can better prepare for adult life. To capture the complexity of psychological wellbeing during adolescent development, this study has focused on using Ryff's model of psychological wellbeing. Moreover, several empirical studies (Forgeard et al., 2011; Huppert and So, 2013; Friedman and Kern, 2014) have emphasized that optimal development during adolescence is based on a multidimensional model and functioning well across multiple psychosocial domains. It is also highlighted that psychological wellbeing during this developmental period is linked to various personal attributes such as autonomy and identity.

### Adolescent's emotional autonomy from parents and wellbeing

Adolescent emotional autonomy from parents is one of the adolescents' most vital developmental tasks. Emotional autonomy requires a recognition of the self as a separate person from one's parents (Blos, 1979). To accomplish this, adolescents must move past the idealized perceptions of their parents and acknowledge that they are regular humans, just like them, who make mistakes and experience problems, too (Levy-Warren, 1999). Blos (1979) emphasized the emotional nature of this process of de-idealization. Similarly, Zimmer-Gembeck and Collins (2003) highlighted how emotional experiences are at the heart of gaining emotional autonomy.

In the family relationship context during adolescence, there are three dimensions associated with autonomy. The behavioral dimension is the first one, which is related to an adolescent's ability to act or perform independently. Cognitive is the second dimension and encompasses attaining self-agency and a sense of competence that empowers the person to decide how to take control of his or her personal life. The third dimension is emotional, and it denotes the evaluation of independence in the form of self-esteem and individuality, including the formation of new emotional bonds that are more egalitarian than those formed during childhood relationships (Parra et al., 2014; Soenens et al., 2017; Reis et al., 2018; Bojanowska and Piotrowski, 2019). Furthermore, emotional autonomy is the interest of the present study among the three aspects of autonomy.

The relationship between emotional autonomy and psychological wellbeing has yet to be clarified. Several authors from the neo-analytic perspective pointed out that emotional distancing from parents as a second process of separation-individuation during adolescence is an essential and vital prerequisite for positive development outcomes (Blos, 1979; Steinberg and Silverberg, 1986). Thus, adolescents become more responsible for their actions and judgments by severing ties to their co-opted parents. In addition, Steinberg and Silverberg (1986) stressed that emotional autonomy is positively associated with health and adjustment during this period and would likely increase during the development. Distancing from parents is a transitional process indicating a time in gaining an optimal sense of individuation, an affirmative view of the self, and an ability to hold its one's own beliefs and judgments firmly. A study conducted by Beyers et al. (2003) pointed out that emotional separation is a healthy and adaptive process experienced by most adolescents; however, a small group of adolescents experienced it in a more negative and conflictive one. Therefore, it would be expected that emotional separation increases continuously during adolescence but provides different outcomes. That said, some authors questioned the need for separation from parents during this developmental period because there are tendencies that moving away does not produce positive development outcomes (Ryan and Lynch, 1989; Fuhrman and Holmbeck, 1995).

After exploring the concept of emotional autonomy, some authors have suggested that the relationship between emotional autonomy and adjustment may vary based on age (Brown et al., 1993; Silverberg and Gondoli, 1996). As a result, they argue that emotional autonomy may have a more positive impact in later adolescence and early adulthood than in earlier years (Beyers and Goossens, 2003). These authors believe that as children develop a more coherent sense of self and their family system adjusts during the initial years of adolescence, their emotional autonomy from their parents may become more positive. However, there is a lack of research into the development and significance of emotional autonomy during early adulthood (Beyers and Goossens, 2003; Kins et al., 2013).

Research examining family relationships during early adulthood has shown that these relationships are influenced by the history of the family relationship in previous years. Therefore, family interactions during young adulthood largely depend on the previous patterns of interaction (Tubman and Lerner, 1994; Thornton et al., 1995). It is important to note, however, that this continuity tends to weaken over time (Belsky et al., 2001). In other words, the evidence of continuity is stronger when comparing the relationships between emerging adults and their parents to those of adolescent-parent relationships than when comparing to the years prior to childhood. Research also suggests that during their third decade of life, young adults tend to gain relative power in their relationships with parents and that family relationships typically improve during these years, with lower rates of conflict (Noack and Buhl, 2005).

The growth of emotional autonomy and its implications for adolescents' psychological wellbeing needs to be more understood. In the study of Steinberg (2001), it has been emphasized that being autonomous may be difficult for adolescents because conflicts during this period over parental distancing concerns are the primary source of disagreements between adolescents and their parents. Moreover, autonomy is often equated with rebellion, and becoming independent is often associated with severing the relationships (parent-adolescent) and distancing itself from his or family (Hill and Holmbeck, 1986). It is also argued that adolescents' emotional autonomy is associated with “self-doubt” and increased feelings of refusal for parents (Steinberg et al., 1994). Conversely, as an alternative to the detachment perspective of emotional autonomy, research has suggested the development of emotional autonomy to develop a sense of individuation and that adolescents can become emotionally autonomous (Grotevant, 1997).

Based on these two opposing results, Beyers et al. (2005) have distinguished between two features of emotional autonomy, including a more adaptive separation and a hostile detachment. Separation has an affirmative implication. It involves “distancing from childhood depictions of parents but is complemented by positive feelings toward them” (Beyers et al., 2005). However, detachment has an adverse connotation. This is vastly influenced by disbelief and a perception of alienation to parents. In this sense, adaptive separation would be connected with the wellbeing of the adolescents and another positive development outcome. In contrast, detachment would be correlated to adolescents' instabilities and negative behavior (Beyers et al., 2005). Together, prior work suggests that aspects of emotional autonomy affect adolescents' psychological wellbeing positively and negatively. Hence, questions and issues remain about the applicability of results within these two constructs.

### Adolescent's identity commitment

Several authors have emphasized that identity formation is one of the most important developmental tasks for understanding adolescents' development. It is defined as the precise construction of the self that is made up of goals, beliefs, and behaviors to which one is firmly committed (Benson et al., 2006). In light of the works of Erikson, Marcia (1980) developed an identity model to improve the conceptualization of Erikson to identity. Marcia's model of identity development is termed as the existence or non-existence of two adolescents' psychological tasks, including exploration, named initially as “crisis,” and another one is commitment (Marcia, 1980). Exploration is the process by which adolescents consider new roles, open up to new possibilities, and try different alternatives based on ideas on standards, skills, values, and goals.

On the other hand, commitment is this study's primary focus. It is defined as different options and actions based on the emerging understanding and evaluation of who one is (Marcia, 1980). Commitments are typically made regarding values, personal limitations, expectations, goals, beliefs, and educational decisions. In this connection, healthy identity commitment is based on personal self-evaluation and driven by emerging self-valuing, not on others' feedback and expectations but on discerning self from others (Coleman, 2011). Thus, acting on self-knowledge and judgment is critical to authentic and healthy identity formation.

Identity commitment is built on multi-identity-related domains. Some studies on this identity construct have identified one overall factor of commitment (Crocetti et al., 2008) or two extensive domains of commitment: personal and ideological (Grotevant and Adams, 1984). This current study takes a slightly different approach by considering the two specific areas of personal identity: interpersonal and career (Meeus et al., 1999). These two domains of identity are fundamental and noticeable for most adolescents (Skorikov and Vondracek, 2011). This study focuses on the appropriate identity commitment of two specific life stages within each domain to determine the role or influence of identity commitments on adolescence.

The first is the career domain, which includes the commitment to study and related educational activities. At school, young people make the first significant decisions. Due to the relevance of educational decisions regarding career opportunities, educational identity is likely an antecedent of career, occupational, or professional identity. The formation of occupational and educational identity is inextricably intertwined (Negru-Subritica et al., 2017).

Second, within the realm of interpersonal identity, as presented first by Grotevant et al. (1982), this study highlights the context of a commitment to a friend or a best friend. They argued that peers provide a context in which young people can explore and develop their sense of self, as well as providing social support and validation. The commitment to a friend or best friend is a particularly salient aspect of peer relationships in adolescence. During this developmental period, friends become increasingly important as individuals navigate the challenges of forming their sense of self and establishing their place within social groups. Research has consistently shown that peer relationships, and specifically close friendships, are critical for adolescent development. Brown (2004) notes that friends become more important in adolescence than they are in childhood, as adolescent begins to rely more heavily on their peers for social and emotional support.

#### Identity and emotional autonomy

Research on the association between emotional autonomy and identity within the frameworks of Erikson and Marcia has been limited. Instead, Kroger and Haslett (1988) proposed that adolescents' identity formation results from the redefinition of parent-child relationships during adolescence, as per the classic separation-individuation theory of Blos (1979). Adolescents' self-confidence and trust in their own possibilities, resulting from a positive outcome of the separation-individuation process, serve as the foundation for structuring psychosocial identity (Josselson, 1988). Numerous studies have focused on linking the family systems paradigm with adolescent development (Perosa et al., 2002). During this transitional period, parents continue to play an important role in the process of identity formation, which, in turn, influences emotional adjustment (Arnett, 2000; Luyckx et al., 2007).

The contradictory findings in empirical studies on the relationship between the move away from parents and identity formation could be attributed to several factors. For instance, the cultural and social context in which the adolescent is embedded could play a crucial role in shaping this relationship (Phinney et al., 2000). Furthermore, the age at which the move away from parents occurs and the level of autonomy granted to the adolescent could be significant moderators of this relationship (Arnett, 2000). Another factor that could impact this relationship is the quality of the parent-adolescent relationship. A positive and supportive relationship between parents and adolescents could facilitate identity formation and the move away from parents, whereas a conflictual and hostile relationship could hinder these processes (Luyckx et al., 2007).

Based on these considerations, this study aims to propose a novel model in which the disengagement from parental ties and the process of identity formation are viewed as two simultaneous processes rather than a prerequisite of each other. The researcher obtained empirical evidence supporting this model through various patterns that linked parental support, identity process, and emotional adjustment. The first pattern indicated a decline in parental support and its association with emotional adjustment as age increased, while the second pattern showed an increase in interpersonal and social commitment with age, and a parallel shift in the relationship between such commitment and emotional adjustment. The reduction in parental support can be interpreted as a separation effect, while the rise in commitments can be seen as an individuation effect. Therefore, the study concluded that these two processes should be regarded as parallel, rather than one being a prerequisite for the other (Meeus et al., 2005).

#### Identity commitment and wellbeing

The commitment and exploration level are expected to influence the positive development of an adolescent significantly. In a study of Berzonsky (2004) among 145 late adolescents, he stresses that those late adolescents with high “self-regard” be likely to have a high level of identity commitment. Furthermore, in the same way, they are more psychologically adjusted than those their counterparts (Meeus et al., 2005). Research on “self-concept clarity” and “self-certainty” have shown that comprehensiveness and clarity of defining oneself are predicted by a high level of individuality (Campbell et al., 1996), meaning a clear sense of identity enables an individual to adapt effectively to difficulties (Erikson, 1968).

Additionally, several studies (Berzonsky, 2004; Luyckx et al., 2007; Pace and Zapulla, 2009) have examined the predictive value of identity processes to all domains of personal satisfaction and found that identity commitment is a predictor of family and satisfaction with self. Meeus et al. (1999) indicate that adolescents with the lowest level of wellbeing tend to possess moratorium status. On the one hand, adolescents reported to have the highest level of wellbeing are those in the foreclosed status. Based on that data, the non-existence of commitment in active exploration debilitates wellbeing. In this regard, it would believe that identity commitment is vital and can be considered the “strength of identity” (Meeus et al., 1999) that improves wellbeing of an individual.

### Identity commitment as moderator

The significant role of identity commitment can go beyond simple associations with emotional autonomy and psychological wellbeing. Although previous studies showed a strong positive connection found between adolescents' emotional autonomy and psychological wellbeing (Beyers et al., 2003; Pace and Zapulla, 2009), it is also expected that individuals vary in “capability” (Beyers et al., 2005). This is highlighted in empirical results, as not all findings showed a substantial and significant effect (Beyers et al., 2005). The more substantial commitment may form a buffer contrary to the debilitating effect of emotional detachment from parents on adolescents' psychological wellbeing (Pace and Zapulla, 2009). Commitment could act as a compass for life at uncertain times. Adolescence with more substantial commitment can cope better with the indices of maladjustment, as they have more explicit goals to guide them.

A study by Pace and Zapulla (2009) shows that the interaction between identity commitment and emotional autonomy played an important role in adolescents' satisfaction with self. The level of self-satisfaction is higher at a high level of detachment when the level of commitment is high. It would explain that if an adolescent think that their withdrawal from parents is ambiguous and resemble a kind of emotional distance, the high level of commitment plays a significant role in adolescents' wellbeing. A higher level of commitment acts as a buffer in developing a satisfying self-image. Therefore, an appropriate confidence level about options on different domains of identity made an adolescent inextricably satisfied with themselves. In line with past studies (Meeus et al., 2005), during adolescence, commitment makes them more aware of themselves and able to establish a well-defined formation of identity from which an adolescent can surpass tasks during development. The previous research emphasizes that this capability could also be a moderator when the perceived move away from parents is ambiguous. This feature can explain how personal resources are important in moderating the track from the perception of poor emotional context to individual maladjustments.

In this connection, this research sought to determine and establish a model on which identity commitment moderates the relationship between emotional autonomy and adolescents' psychological wellbeing rather than separation from parents as a prerequisite of identity formation or identity formation as a prerequisite of emotional autonomy because these two developmental tasks are considered different tasks in adolescent development (Meeus et al., 2005). As such, in this study, identity commitment is conceptualized as a moderator in the relationship between emotional autonomy and psychological wellbeing.

### Conceptual and theoretical framework

The developmental tasks theory, often associated with the work of psychologist Havighurst (1952), emphasizes the importance of specific tasks or milestones that individuals must accomplish at different stages of their lives to achieve optimal development. In the context of adolescence, two critical developmental tasks are autonomy and identity formation. These tasks play a significant role in the wellbeing of middle and late adolescents. Emotional autonomy refers to an individual's ability to perceive oneself as independent and self-assured, with a strong sense of individuality, and to establish emotional connections that are more balanced than those experienced during childhood relationships while identity commitment refers to the degree to which an individual has made a commitment to their personal identity and it suggests that identity commitment is an important factor in determining psychological wellbeing. When individuals have a strong commitment to their personal identity, they are more likely to experience a sense of purpose and meaning in their lives, which can promote psychological wellbeing.

**Figure 1:**
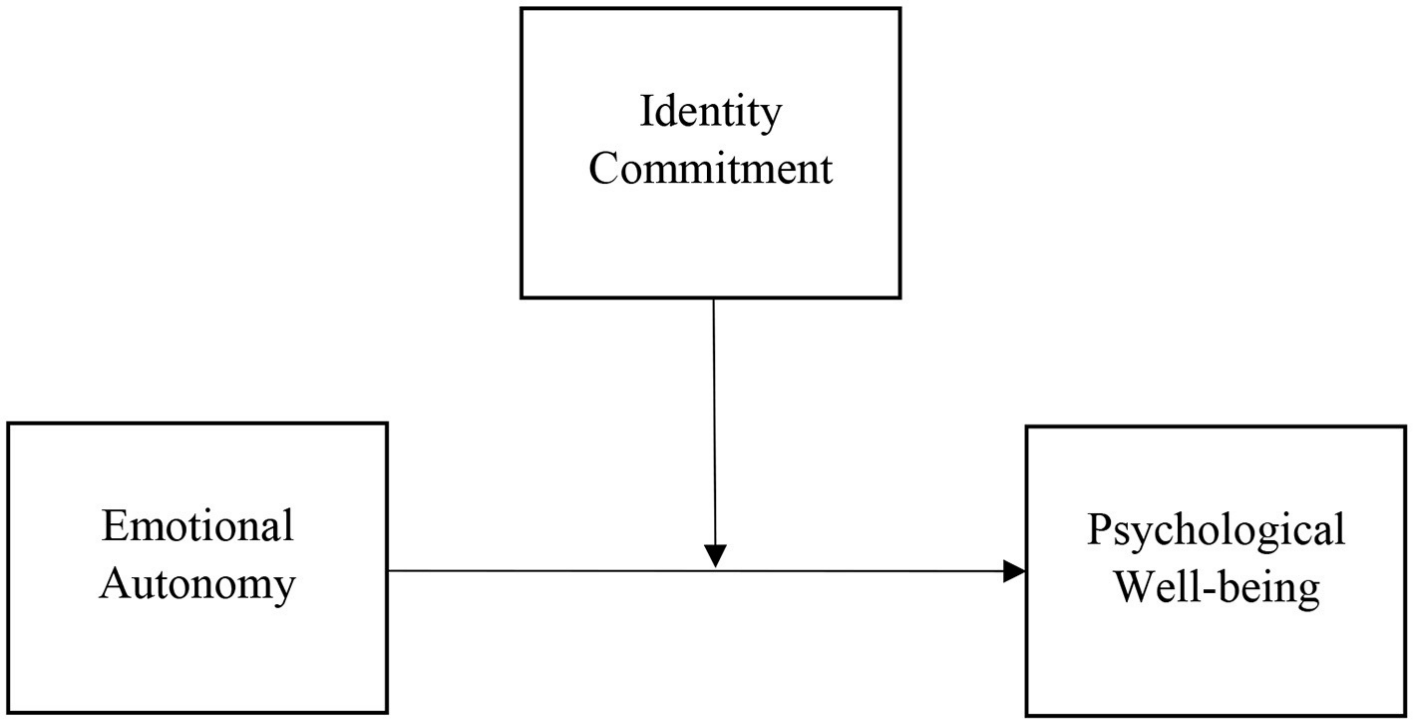
Model representing the moderating role of identity commitment to the relationship of emotional autonomy and psychological wellbeing.

Finally, it proposes that the relationship between emotional autonomy and psychological wellbeing is moderated by the strength of an individual's identity commitment (see Figure 1). Specifically, it suggests that a strong commitment to one's identity can have a positive impact on the relationship between emotional autonomy and psychological wellbeing. Therefore, it can provide a valuable framework for understanding the complex relationships between emotional autonomy, identity commitment, and psychological wellbeing. By examining these relationships through the lens of this theory, researchers can gain a deeper understanding of the factors that contribute to optimal psychological functioning and wellbeing. As such, this present study tries to understand whether (1) emotional autonomy predicts psychological wellbeing, (2) assesses how identity commitment influences psychological wellbeing, and (3) how identity commitment moderates how emotional autonomy and psychological wellbeing work together.

Three cross-sectional hypotheses were carried out in this paper. First, it is hypothesized that emotional autonomy predicts decrease in psychological wellbeing (hypothesis 1). Further, identity commitment positively affects psychological wellbeing (hypothesis 2). Finally, the impact of emotional autonomy on psychological wellbeing will decrease when identity commitment is present (buffering effect).

## Methods

### Research design

In the current study, a descriptive-correlational research design was employed. Correlational research is a type of non-experimental research that enables the prediction and explanation of relationships among variables. As outlined by Creswell and Guetterman (2018), important characteristics of correlational research include examining the correlation between two or more variables, collecting data at a single point in time, utilizing all scores on a continuum, obtaining at least one score for each variable using an appropriate correlational statistic, and drawing an interpretation to arrive at a conclusion based on the result of the statistical test.

### Participants

The study utilized an online sample size calculator to determine the total number of participants needed. With a confidence level of 95%, a margin of error of 5%, a population proportion of 50%, and an estimated adolescent population of 19.8 million (according to the Philippine Statistics Authority in 2020), the calculator suggested that 385 participants were needed. However, the researcher purposively selected 454 participants to ensure adequate representation across various demographic categories. The study included adolescents between the ages of 15 and 19 years, as defined by the World Health Organization (WHO) as the middle to late adolescent stage. The average age of the participants was 17.96 years old. In terms of gender and sexual orientation, there were 108 male participants, 323 female participants, 14 who identified as bisexual, seven as lesbians, and two as gay. Regarding family arrangement, the majority of the participants (340) came from two-parent households, while 86 were living only with their father and 28 with their mother. Most of the participants' parents had completed high school as their highest level of education, and the estimated annual family income ranged from 200,001 to 300,000 Philippine pesos. Overall, these inclusion criteria ensured that the sample was diverse and representative of the adolescent population in the Philippines, which enhances the generalizability of the study findings to this population.

### Instruments

At the beginning of the questionnaire, adolescent participants responded to questions about their age, sex, and data about their families.

#### Wellbeing

Ryff's Psychological Wellbeing Scale (PWB; Ryff, 1989) was used to assess adolescents' wellbeing. This is a multidimensional measure of psychological wellbeing with 42 items and six subscales anchored from Ryff's Multidimensional Model of Psychological Wellbeing. The PWB scales are designed to assess positive psychological functioning in six theoretically distinct domains (Ryff, 1989): self-determination and independence (autonomy), selection or creation of contexts that suit personal needs and values (environmental mastery), openness to new experiences and opportunities for self-improvement (personal growth), having warm, satisfying, trusting relationships (positive relations with others), finding meaning in one's current and past activities (purpose in life), and having positive attitudes toward the self and one's past (self-acceptance): self-acceptance (seven items; e.g., “In general, I feel confident and positive about myself”), purpose in life (seven items; e.g., “I am an active person in carrying out the plans I set for myself at work”), personal growth (seven items; e.g., “I have the sense that I have developed a lot as a person through my work”), positive work relations with others (seven items; e.g., “I enjoy personal and mutual conversations with the people I work with”), environmental mastery (seven items; e.g., “I am quite good at managing the many responsibilities of my daily work life”), autonomy (seven items; e.g., “My work decisions are not usually influenced by what everyone else at work is doing”). Participants responded on a scale from 1 (strongly disagree) to 6 (strongly agree). Responses were totaled for each of the six subscales (about half of the responses were reverse scored) and, form each subscale, high scores indicated that the respondent has a mastery of that area in his or her life, whereas low scores showed that the respondent struggles to feel comfortable with that particular area. A total PWB score was calculated by adding all items of six dimensions. Using Cronbach's alpha, the reliability of Ryff's Psychological Wellbeing Scale is 0.85.

#### Emotional autonomy

The Emotional Autonomy Scale (EAS; Steinberg and Silverberg, 1986) was used to assess the emotional autonomy of the adolescent participants. The Emotional Autonomy Scale consisted of 20 items that were answered on a 4-point Likert-type scale ranging from strongly disagree to strongly agree. An increase in the score on the scale was assumed to be an indicator of high emotional autonomy. The measure consisted of four subscales, two assessing affective characteristics of autonomy (Individuation from parents and Non-dependency on parents) and two quantifying cognitive facets (Parental de-idealization and Perceived parents as people). Sample items are: “I go to my parents for help before trying to solve a problem myself” (reverse coded: Nondependency on parents). “My parents know everything there is to know about me” (reverse coded: Individuation). “I have often wondered how my parents act when I am not around” (Perceives parents as people). “My parents and I agree on everything” (reverse coded: Deidealization). Using Cronbach's alpha, the reliability of Emotional Autonomy Scale is 0.74.

#### Identity commitment

The Utrecht-Management of Identity Commitments Scale (U-MICS; Crocetti et al., 2010) was used to measure adolescents' identity-related process of commitment, which included commitments to education and friends on which these domains are highly salient for a vast majority of adolescents (Skorikov and Vondracek, 2011). The U-MICS consisted of 13 items with a response scale ranging from 1 (completely untrue) to 5 (completely true). The current study assessed the identity dimensions in one ideological domain (education) and one interpersonal domain (friends). Each item is presented once for the ideological domain and once for the interpersonal domain, for a total of 26 items. Specifically, ten items measure commitment; ten assess in-depth exploration, and six items tap reconsideration of commitment. Sample items include: “My education/friend gives me certainty in life” (commitment), “I think a lot about my education/friend” (in-depth exploration), and “I often think it would be better to try to find a different education/friend” (reconsideration of commitment). For each identity dimension, I will sum the responses across the two domains (Crocetti et al., 2008). Using Cronbach's alpha, the reliability of the U-MICS is 0.88.

### Procedures

1. Collaboration with school divisions. The researchers first collaborated with the School Divisions of Nueva Ecija, Science City of Munoz, San Jose City, and Cabanatuan City to conduct the study. This involved getting permission from the school authorities to conduct the survey and obtaining their assistance in distributing the questionnaires.
2. Selection of participants. To ensure a representative selection of participants, the researchers made sure that each of the four school divisions included both public and private schools. The study focused on classes from grades 10–12 in each school.
3. Ethical standards. The researchers adhered to ethical standards set by De La Salle University—Manila.
4. Informed Consent. The participants' authorized caregivers were asked to provide consent, and the participants themselves provided assent to participate in the study. This ensured that the participants and their caregivers were fully informed about the study and gave their permission to participate voluntarily.
5. Questionnaire distribution: The researchers provided the participants with questionnaires to be completed at home. The questionnaires were designed to collect self-report data on identity commitment, emotional autonomy, and wellbeing. The participants were also provided with socio-demographic information such as age, sex, and family data.
6. Data collection: The data collection was anonymous to ensure that the participants could answer the questions honestly and without fear of repercussions. The researchers collected the completed questionnaires from the participants' teachers at the school or through other designated drop-off locations.
7. Data summary: After collecting the questionnaires, the researchers compiled and summarized the data. The data was analyzed using quantitative methods, and statistical software was used to determine any associations among the variables.

### Data analysis

The data were manually encoded, and strict measures were taken to ensure the integrity of the data by verifying if the respondents met the inclusion criteria. Additionally, demographic information of participants who refused to join was documented to analyze potential selection biases in the dropout rate. Data cleaning was given utmost importance and was carried out meticulously, including the handling of outliers, problematic cases, missing values, and normality.

For the actual analysis, the data were examined in accordance with the purpose of the study. Multiple linear regression analysis was used to draw inferences related to hypotheses one and two. To investigate the extent to which identity commitment can moderate the relationship between emotional autonomy and wellbeing, moderation analysis using Model 1 was performed. The first two objectives were analyzed using the software SPSS v. 28, while SPSS—PROCESS was used for the third objective.

### Ethics

The present study was conducted with a commitment to ensuring the utmost honesty and impartiality in all research procedures. The researcher duly recognized that obtaining informed consent from participants was contingent upon their willingness to engage in a collaborative working relationship. In this regard, the researcher took reasonable measures to ensure that the information provided to participants was fully comprehensible and accessible, including translating consent forms into languages or dialects that participants could understand. It is important to note that obtaining written informed consent from prospective participants constitutes more than mere signature on a form. Rather, the consent document served as a framework for the researcher's verbal explanation of the study, facilitating a meaningful and informative exchange between researcher and participant. While the participant's signature provided documentation of their agreement to participate in the study, it was only one aspect of the informed consent process. The consent document should never serve as a substitute for a comprehensive discussion of the research aims and procedures.

In order to be effective, the informed consent process necessitates the provision of ample subject information concerning the study, allowing sufficient opportunity for the participant to consider all options, and responding to any questions or concerns they may have. It is equally important to ensure that the participant has fully comprehended this information before obtaining their voluntary agreement to participate. Additionally, ongoing provision of information and support is essential throughout the duration of the study, with the researcher and participant engaging in open and transparent communication. In summary, the researchers adhered to ethical standards set by De La Salle University—Manila.

## Results

This section presents the results of the data analysis conducted in this study. Firstly, the descriptive statistics and inter-correlations of all study variables are presented. Secondly, the results of the Conditional Process Analysis are presented, which will shed light on the moderating role of identity commitment in the relationship between emotional autonomy and psychological wellbeing.

Data were gathered from a sample of middle to late adolescents (*N* = 454) aged 15 to 19 (*M* = 17.96, *SD* = 0.934). Their level of emotional autonomy ranged from 1.85 to 3.75 (*M* = 2.64, *SD* = 0.321); ratings of identity commitment ranged from 1.77 to 5.00 (*M* = 3.49, *SD* = 0.460); and level of psychological wellbeing ranged from 2.24 to 5.43 (*M* = 3.87, *SD* = 0.465).

Table 1 presents the inter-correlation of age, emotional autonomy, identity commitment, and psychological wellbeing. Data were analyzed using Pearson Moment Correlation Coefficient. The results showed that psychological wellbeing is significantly correlated with emotional autonomy, and identity commitment. Psychological wellbeing is positively correlated to identity commitment. However, psychological wellbeing is negatively correlated to emotional autonomy which indicates that at a higher level of emotional autonomy, there is a decrease in psychological wellbeing. And on the other hand, emotional autonomy is also negatively correlated with identity commitment. However, age is inversely correlated to identity commitment.

**Table 1:** Descriptive statistics and correlation of the variables.

**Variables**	**Mean**	**SD**	**1**	**2**	**3**	**4**
Age	17.96	0.934		0.048	−0.102^*^	0.014
Emotional autonomy	2.64	0.321			−0.211^**^	−0.267^**^
Identity commitment	3.49	0.460				0.203^**^
Psychological wellbeing (PWB)	3.87	0.465				

^*^Correlation is significant at 0.05 level. ^**^Correlation is significant at 0.01 level.

Table 2 shows the impact of age, emotional autonomy and identity commitment to psychological wellbeing. The *R*^2^ of 0.10 revealed that the predictors explained 10% variance in the outcome variable with *F*_(3, 450)_, 15.82, *p* < 0.000. The findings revealed that age does not significantly predict psychological wellbeing (β = 0.041, *p* < 0.363) whereas emotional autonomy negatively predict psychological wellbeing (β = −0.235, *p* < 0.000), and identity commitment positively predict psychological wellbeing (β = 0.158, *p* < 0.001).

**Table 2:** Predicting psychological wellbeing using multiple linear regression analysis.

**Variables**	**Beta**	**SE**	**95% CI**		**β**	***p***
			**LL**	**UL**		
Age	0.020	0.022	−0.024	0.065	0.041	0.362
Emotional autonomy	−0.340	0.066	−0.471	−0.210	−0.235	0.000
Identity commitment	0.159	0.047	0.068	0.251	0.158	0.001

^**^p ≤ 0.01.

### Identity commitment as moderator of emotional autonomy and psychological wellbeing

A conditional process analysis using Model 1 of PROCESS (Hayes, 2022) was done to test the moderating effect of identity commitment in the relationship between emotional autonomy and the psychological wellbeing of Filipino middle and late adolescents. Table 3 shows that the overall model fit is significant which means that identity commitment has moderating effects on emotional autonomy and psychological wellbeing [*F*_(3, 450)_ = 18.98, *p* < 0.0000, *r*^2^ = 0.112)]. This means that the effect of emotional autonomy on psychological wellbeing can depend on the level of identity commitment.

**Table 3:** Overall model fit of emotional autonomy and psychological wellbeing as moderated by identity commitment.

	***r***	***r^2^***	**MSE**	***F***	***df1***	***df2***	***p***
Model	0.335	0.112	0.193	18.98	3	450	0.0000

^**^p ≤ 0.01.

**Figure 2:**
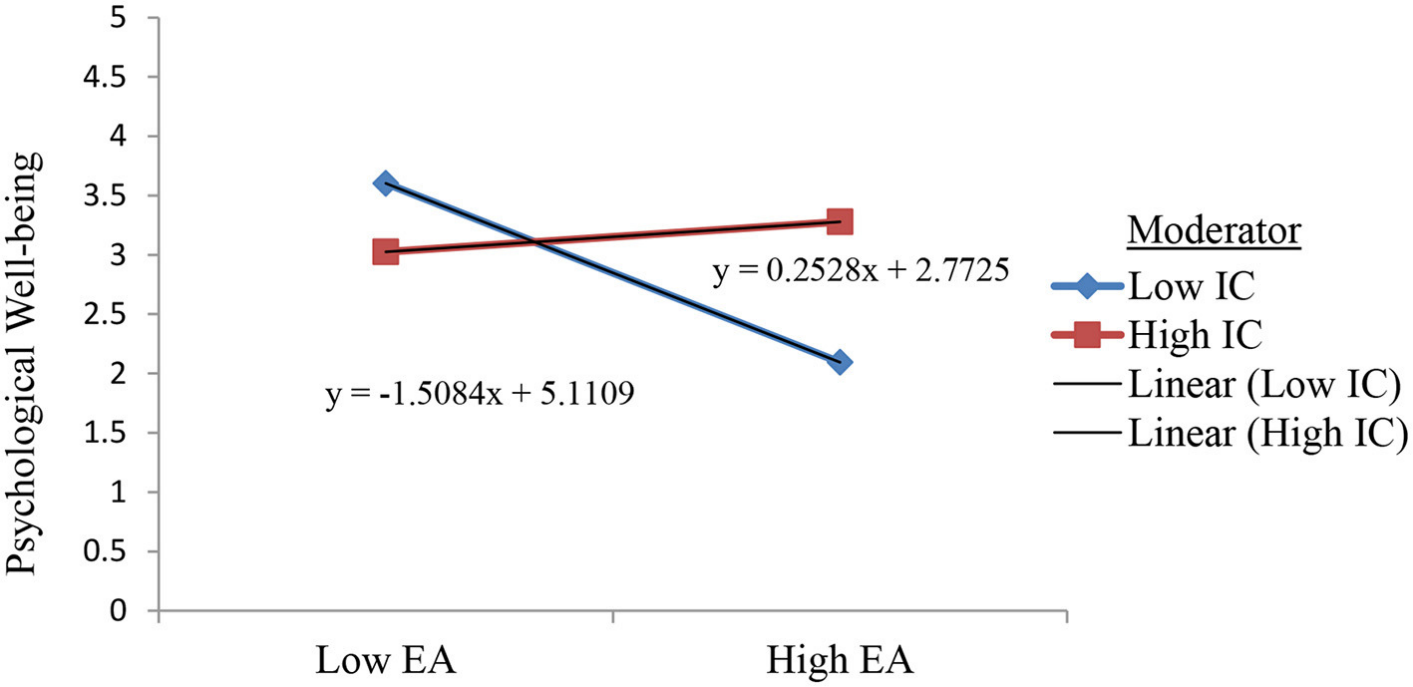
Interaction plot for identity commitment as a moderator between emotional autonomy and psychological autonomy. EA, emotional autonomy; IC, identity commitment.

Looking further into the model, Table 4 shows the interactions within the model. It is shown that emotional autonomy is negatively predicted psychological wellbeing (*B* = −0.3139, *SE* = 0.0662, *p* = 0.0000). On the other hand, identity commitment served as a positive predictor (*B* = 0.1517, *SE* = 0.0459, *p* = 0.0010). The result also shows that identity commitment moderated the relationship between emotional autonomy and psychological wellbeing (*B* = 0.4403, *SE* = 0.1435, *p* = 0.0023). This interaction is illustrated in Figure 2.

**Table 4:** Moderating effect of identity commitment on the relationship between emotional autonomy and psychological wellbeing.

**Variables**	***B***	**SE**	***t***	**95% CI**		***p***
				**LL**	**UL**	
Emotional autonomy	−0.3139	0.0662	−4.7413	−0.4439	−0.1838	0.0000
Identity commitment	0.1517	0.0459	3.3040	0.0615	0.2420	0.0010
EA x IC	0.4403	0.1435	3.0689	0.1583	0.7223	0.0023

Table 5 displays the conditional effect, it reveals that emotional autonomy negatively predicted psychological wellbeing when identity commitment is low (*B* = −0.516, *SE* = 0.0876, *p* = 0.0000) or average (*B* = −0.3139, *SE* = 0.662, *p* = 0.0000). However, at high level of identity commitment, emotional autonomy did not predict psychological wellbeing (*B* = −0.1112, *SE* =0.09991, *p* = 0.2624).

**Table 5:** Conditional effect of identity commitment on the relationship between emotional autonomy and psychological wellbeing.

**Identity commitment**	***B***	**SE**	***t***	**95% CI**		***p***
				**LL**	**UL**	
−0.4603	−0.516	0.0876	−5.8982	−0.6886	−0.3444	0.0000
0.0000	−0.3139	0.0662	−4.7413	−0.4439	−0.1838	0.0000
0.4603	−0.1112	0.0991	−1.1221	−0.3059	0.0835	0.2624

## Discussion

The aim of the present study was to investigate the association between emotional autonomy, identity commitment, and psychological wellbeing in adolescents. Specifically, the study focused on examining the independent and combined contributions of emotional autonomy and identity commitment to psychological wellbeing.

### Emotional autonomy and wellbeing

The findings indicated that emotional autonomy and identity commitment were associated with psychological wellbeing in different ways. Interestingly, emotional autonomy was found to predict psychological wellbeing inversely, suggesting that the ability of adolescents to let go of the unrealistic images of their parents may be related to the decline in middle and late adolescents' psychological wellbeing. This inverse prediction could be related to the challenges that adolescents face in achieving emotional autonomy while still maintaining healthy relationships with their parents. Adolescents who have high levels of emotional autonomy may feel disconnected from their parents or experience conflicts in their relationships, leading to negative outcomes (Melendro et al., 2020). This finding contrasts with previous research that viewed emotional separation from parents as a necessary condition for individuation and normal adolescent development (Meeus et al., 2005). Though emotional distancing from parents is a vital developmental process for individuation to move forward because it increase subjective sense of her or his independence particularly in relation to parents, it may initially leave middle and late adolescents emotionally vulnerable and empty, leading to a decline in psychological wellbeing and to some degree of maladjustment. Ingoglia et al. (2011) and Sugimura et al. (2020) both emphasize the negative impact of emotional separation on psychosocial adjustment and problem behavior, with the latter also distinguishing between emotional separation and detachment as distinct dimensions of the parent-adolescent relationship. These studies collectively suggest that while emotional separation may not be a necessary condition for normal adolescent development, it can have significant implications for adolescents' wellbeing. Emotional detachment does not appear to aid in developing agentic capacities and volitional functioning during adolescence hence, it is not positive indicator with psychological wellbeing or a greater sense of self (Van Petegem et al., 2013).

The Havighurst theory of developmental tasks emphasizes the importance of achieving emotional autonomy during adolescence. This process involves establishing physical and emotional boundaries, developing a sense of agency and autonomy in decision-making, and maintaining a positive relationship with parents while also achieving independence. It is possible also that the inverse relationship between emotional autonomy and psychological wellbeing is explained by the separation-individuation process. According to the separation-individuation, emotional autonomy is achieved through a gradual process of separation from the parent and the development of a unique sense of self. This process involves a number of developmental tasks, including: establishing physical and emotional boundaries between the self and others (differentiation); developing a sense of agency and autonomy in decision-making (individuation) maintaining a positive relationship with parents while also achieving independence (separation). It is possible that adolescents who have difficulty with the separation-individuation process may struggle to achieve emotional autonomy and experience negative outcomes as a result. As Kins et al. (2013) found, when a good equilibrium between individuation and separation is not established, two forms of poor separation-individuation could manifest: “dysfunctional dependence,” a propensity to overly pursue closeness with others, or “dysfunctional independence,” a tendency to be intensely engaged with individuality and to reject various forms of contact. The adolescents in this study who exhibit higher levels of emotional autonomy from their parents may not have reached this delicate balance between separation and individuation, leading to lower psychological wellbeing. Perhaps because of this, Kins et al. (2013) would classify them as having “dysfunctional independence.”

Furthermore, in the context of this research findings, it is possible that the inverse relationship between emotional autonomy and psychological wellbeing may be more pronounced in collectivist cultures. For example, adolescents who are more emotionally autonomous in a collectivist culture may be viewed as deviant or disobedient, leading to negative social and psychological outcomes. This reflect of the Filipino notion of “*kapwa”* (Enriquez, 1977), which explains the Filipino sensitivity to others that Filipino adolescent's actions and choices are influenced not only by personal interests, but also by how their decisions and behaviors impact others. As Meeus et al. (2005) emphasized that emotional autonomy may be more emphasized in individualistic cultures. Keeping close emotional bonds with parents is likely a prerequisite for good development in more collectivist societies, such as the Philippines.

### Identity commitment and wellbeing

Identity commitment positively predicts psychological wellbeing, indicating that the adolescent's search for their identity could be associated with a higher level of psychological wellbeing. According to Havighurst's developmental tasks theory, adolescents face numerous developmental tasks, one of which involves establishing a sense of identity. This finding suggests that when adolescents commit to a clear sense of identity, it positively influences their psychological wellbeing. Havighurst posited that successfully navigating these tasks contributes to healthy development. Therefore, as adolescents engage in the exploration and resolution of their identity, their commitment to a chosen identity is likely to enhance their overall psychological wellbeing. This association underscores the significance of identity formation in fostering positive mental health outcomes during adolescence, aligning with Havighurst's framework that emphasizes the importance of accomplishing developmental tasks for optimal growth and adjustment.

Moreover, the positive relationship between identity commitment and psychological wellbeing can be explained by several mechanisms. First, identity commitment provides a sense of continuity and stability in the face of life's challenges. Adolescents who are committed to their identities are better able to weather setbacks and difficulties because they have a strong sense of self and a clear direction in life. Second, identity commitment promotes positive psychological adjustment by providing a source of self-esteem and self-worth. Adolescents who are committed to their identities are more likely to feel a sense of pride and accomplishment in their achievements, which can contribute to greater feelings of wellbeing. Finally, identity commitment promotes social integration by facilitating positive relationships with others who share similar values and goals. It seems easier to allow middle and late adolescents to reach their full potential when they have a clearer idea of who they are. Data showed that identity commitment predicted adolescent psychological wellbeing, consistent with earlier studies (Berzonsky, 2004; Karas and Cieciuh, 2018) regarding the contribution of identity to psychological wellbeing. In the process of developing personal goals connected to wellbeing, commitment has a definite role.

Additionally, research has suggested that identity commitment may be particularly important during adolescence, a time when individuals are exploring different aspects of their identity and making important decisions about their future. Adolescents who are able to establish a strong sense of identity during this period are more likely to experience positive outcomes, such as greater self-esteem and less anxiety. Prior studies have shown that commitment is an adaptive process that is positively predicts with wellbeing (Meeus, 2011; Karas and Cieciuh, 2018). The results of this present study have also verified empirical studies (Dimitrova et al., 2017; Karas and Cieciuh, 2018) that show the beneficial impact of identity commitment in reaching wellbeing.

In line with this, identity commitment may promote positive psychological adjustment by providing a sense of coherence and integration in the face of life's challenges. Adolescents who are committed to their identities are better able to integrate new experiences into their sense of self and to make sense of their world in a meaningful way. This, in turn, may contribute to greater feelings of wellbeing. This supports the idea that, for middle and late adolescents, having a solid sense of identity is crucial when pursuing wellbeing. Adolescents may better distinguish themselves from others as independent individuals and respect their boundaries if they have a greater understanding of who they are. Identity commitment may promote social integration by facilitating positive relationships with others who share similar values and goals. Adolescents who are committed to their identities may be more likely to seek out and form relationships with others who are similarly committed, which can provide a sense of belonging and social support. Finally, adolescents with a stronger sense of who they are will likely perform better in many aspects of life (Umaña-Taylor, 2018).

### Identity commitment as moderator

Apart from investigating the separate impacts of emotional autonomy and identity commitment on psychological wellbeing, the current study also explored the interplay between emotional autonomy and psychological wellbeing, considering the level of identity commitment. The results of the study demonstrated that the association between emotional autonomy and psychological wellbeing is not straightforward but rather depends on the level of identity commitment. When identity commitment is low or average, emotional autonomy does have a negative impact on psychological wellbeing. Specifically, when identity commitment is low, emotional autonomy significantly predicts lower psychological wellbeing. Similarly, when identity commitment is at an average level, emotional autonomy also negatively predicts psychological wellbeing. This means that individuals who have lower levels of commitment to their identity and higher emotional autonomy are more likely to experience lower psychological wellbeing. However, when identity commitment is high, emotional autonomy does not significantly predict psychological wellbeing. In other words, when individuals have a strong sense of commitment to their identity, their level of emotional autonomy does not have a significant effect on their psychological wellbeing.

Let us first consider the situation when identity commitment is at low or average level, emotional autonomy negatively predicts psychological wellbeing. This means that individuals who have lower or average level of identity commitment and higher emotional autonomy are more likely to experience lower psychological wellbeing. In this situation, adolescents may feel a strong desire to be independent and separate from their parents, but they may also lack a clear sense of their own identity. This can lead to feelings of confusion, and uncertainty, which can negatively impact their psychological wellbeing. Without a clear sense of who they are and what they stand for, adolescents may struggle to navigate the challenges of adolescence, and this may make them more vulnerable to negative outcomes.

Adolescents who exhibit lower levels of emotional autonomy and weaker commitment to their identity may experience a reduced sense of control over their lives and diminished confidence in achieving their goals. Consequently, this can lead to a decrease in psychological wellbeing. Furthermore, the increased emotional detachment and lower level of identity commitment are not adaptive as they are less associated with a sense of volition. This suggests that when adolescents perceive their withdrawal from parents as ambiguous and characterized by emotional distance, the lack of commitment to their identity plays a detrimental role in their psychological wellbeing. A reduced level of commitment impedes the development of a fulfilling self-image, leading to lower levels of self-confidence among adolescents. Consistent with previous research (Meeus et al., 2005), during adolescence, when commitment is hindered, individuals may struggle with self-awareness and fail to establish a well-defined identity formation. Consequently, this can hinder their ability to navigate developmental tasks successfully. This characteristic highlights the significance of an individual's personal resources in influencing various aspects, ranging from perceiving a negative emotional environment to experiencing personal maladjustments.

Furthermore, it is also important to note that these findings may be influenced by cultural values regarding independence and interdependence. In cultures that place a high value on independence, such as Western cultures, emotional autonomy may be seen as a positive thing, and adolescents who are both emotionally autonomous and committed to their identity may experience greater psychological wellbeing. In contrast, in cultures that place a high value on interdependence, such as collectivist cultures, emotional autonomy may be less strongly associated with positive outcomes, particularly when accompanied by low levels of identity commitment.

However, where identity commitment is high, emotional autonomy does not significantly predict psychological wellbeing. This means, if individuals possess a strong commitment to their identity, their level of emotional autonomy does not significantly impact their psychological wellbeing. In this circumstance, identity commitment does not function as a moderator, it enables adolescents to explore their own interests and preferences while experiencing a sense of disconnection from their parents, but it does not hamper its negative effect to their ability to navigate challenges.

### Theoretical implication

Drawing upon Havighurst's Developmental Tasks Theory, this finding underscores the intricate interplay between emotional autonomy, identity commitment, and psychological wellbeing during adolescence. According to Havighurst, adolescents must achieve autonomy in emotional expression as part of their developmental tasks. However, this study suggests that the relationship between emotional autonomy and psychological wellbeing is contingent upon the level of identity commitment. When identity commitment is low or average, emotional autonomy exhibits a detrimental effect on psychological wellbeing. Specifically, at lower levels of identity commitment, emotional autonomy significantly predicts lower psychological wellbeing, and a similar negative impact is observed at average levels of identity commitment. This nuanced understanding aligns with Havighurst's theory, highlighting the importance of navigating various developmental tasks, including achieving emotional autonomy, within the context of a secure and committed identity to promote positive psychological outcomes during adolescence.

### Practical implication

The findings of the study suggest that emotional autonomy and identity commitment play significant roles in adolescent psychological wellbeing. The implication of these findings is that adolescent psychologists and counselors need to be aware of the potential impact of emotional autonomy and identity commitment on adolescents' psychological wellbeing. They should pay attention to identifying adolescents who may be at risk of low psychological wellbeing due to low identity commitment and high emotional autonomy. Interventions can then be designed to increase identity commitment and decrease emotional autonomy in these adolescents.

Moreover, the findings also suggest that there is a need for a balance between emotional autonomy and identity commitment. High levels of both emotional autonomy and identity commitment were linked to higher levels of psychological wellbeing, indicating that a balance of these two factors is crucial for optimal psychological wellbeing. Adolescent psychologists and counselors can use this information to guide interventions aimed at fostering a balance between emotional autonomy and identity commitment.

Overall, the study's findings can help guide adolescent psychologists and counselors in understanding and addressing psychological wellbeing issues among adolescents. It highlights the importance of emotional autonomy and identity commitment in adolescent development and suggests ways to promote optimal psychological wellbeing.

### Limitations and recommendations

While the current study provides valuable insights into the complex relationships between identity formation, emotional autonomy, and psychological wellbeing in adolescents, it is important to acknowledge its limitations. The cross-sectional design of the study limits our ability to make causal inferences about the relationships between these variables. Therefore, it is imperative that future research takes a longitudinal approach, following adolescents from adolescence to adulthood, to better understand the developmental trajectories of these variables. In addition, it would have been much richer and more opportune to complete the research with interviews and focus groups, using a mixed method in this sense.

Additionally, the current study relied solely on self-report measures, which may not accurately reflect the reality of the participants' experiences. To address this limitation, future studies should gather information from multiple sources, such as parents and teachers, to obtain a more comprehensive understanding of the relationships between these variables.

Despite these limitations, the findings of the current study contribute significantly to our understanding of adolescent development and wellbeing, particularly from a cultural standpoint. However, there is still much to learn about the complex interplay between identity formation, emotional autonomy, and psychological wellbeing in adolescents. It is crucial that future research continues to explore these relationships in greater depth, employing rigorous research methods and a diverse range of participants to ensure the generalizability of findings. Ultimately, a better understanding of these variables will have important implications for the promotion of healthy adolescent development and wellbeing.

## Conclusion

1. The research findings suggest that in middle and late adolescence, a decrease in emotional autonomy is connected to a decrease in psychological wellbeing. This implies that the capacity to establish independence from parents or let go of unrealistic parental expectations may be tied to a reduction in mental health during this developmental stage.
2. The findings further showed that a moderate and positive correlation exists between identity commitment and psychological wellbeing. This implies that adolescents who have a strong sense of their identity may enjoy better overall wellbeing. This highlights the significance of promoting adolescents to delve into their values, interests, and aspirations, while offering them support and guidance along the way.
3. The results of the study demonstrated that emotional autonomy has a negative impact on psychological wellbeing when identity commitment is low or average. In particular, individuals with a low level of identity commitment experience a significant decrease in psychological wellbeing due to emotional autonomy. Additionally, when identity commitment is at an average level, emotional autonomy also contributes to a decline in psychological wellbeing.
4. The research indicated that individuals with a strong level of identity commitment are not significantly affected by emotional autonomy in terms of psychological wellbeing. This implies that when individuals are deeply committed to their identity, their emotional autonomy does not have a notable impact on their psychological wellbeing.

## Data availability statement

The raw data supporting the conclusions of this article will be made available by the authors, without undue reservation.

## Ethics statement

The studies involving humans were approved by De La Salle University Ethics Board. The studies were conducted in accordance with the local legislation and institutional requirements. Written informed consent for participation in this study was provided by the participants' legal guardians/next of kin. Written informed consent was obtained from the individual(s), and minor(s)' legal guardian/next of kin, for the publication of any potentially identifiable images or data included in this article.

## Author contributions

WR: Writing – original draft. HY: Writing – review & editing.
